# *Chuanzhitongluo* capsule ameliorates microcirculatory dysfunction in rats: Efficacy evaluation and metabolic profiles

**DOI:** 10.3389/fphar.2022.1011333

**Published:** 2022-10-07

**Authors:** Yuanfang Sun, Guoliang Cheng, Lijing Du, Yu Gan, Bing Li, Shikai Yan, Mingguo Shao, Huizi Jin, Shasha Li

**Affiliations:** ^1^ The Second Clinical College of Guangzhou University of Chinese Medicine, Guangzhou, China; ^2^ School of Pharmacy, Shanghai Jiao Tong University, Shanghai, China; ^3^ State Key Laboratory of Dampness Syndrome of Chinese Medicine, The Second Affiliated Hospital of Guangzhou University of Chinese Medicine, Guangzhou, China; ^4^ State Key Laboratory of Generic Manufacture Technology of Chinese Traditional Medicine, Lunan Pharmaceutical Group Co.,Ltd, Linyi, China; ^5^ School of Traditional Chinese Medicine, Beijing University of Chinese Medicine, Beijing, China

**Keywords:** chuanzhitongluo capsule, microcirculatory dysfunction, laser speckle contrast imaging, efficacy outcome, mechanism exploration

## Abstract

**Background:** Ischemic stroke is a leading cause of mortality and disability worldwide. Microcirculatory dysfunction is the foremost hindrance for a good clinical prognosis in ischemic stroke patients. Clinical researches show that *Chuanzhitongluo* capsule (CZTL) has a curative effect during the recovery period of ischemic stroke, which contributes to a good prognosis. However, it is not known whether CZTL treats ischemic stroke by ameliorating microcirculation dysfunction.

**Objective:** In this study, we investigated the influence of CZTL on microcirculation and its underlying mechanism.

**Methods:** A rat model of acute microcirculatory dysfunction was established by stimuli of adrenaline and ice water. The microcirculatory damage in model rats and the efficacy of CZTL were assessed by detecting laser speckle contrast imaging, coagulation function, hemorheology, vasomotor factor and microcirculation function. The potential mechanism of CZTL action was explored by the untargeted metabolomic analysis based on ultra-performance liquid chromatography-quadrupole-time of flight-mass spectrometry.

**Results:** Laser speckle contrast imaging showed that model rats suffered low perfusion in ears, feet and tails, and CZTL treatment increased microcirculatory blood flow. Coagulation function detection results showed that CZTL diminished the reduction of thrombin time, prothrombin time, activated partial thromboplastin time and the elevated fibrinogen level caused by acute microcirculatory dysfunction. Furthermore, CZTL could recover the increased blood viscosity as well as the abnormal vasomotor and microcirculation function in rats with acute microcirculatory dysfunction. Metabolomics analysis indicated that CZTL might regulate sphingolipid metabolism and arachidonic acid metabolism to exert protective effects on microcirculation.

**Conclusion:** These results elucidated that CZTL was highly effective against microcirculatory dysfunction and its potential mechanisms related with the modulation of sphingolipid and arachidonic acid metabolic pathways. The present study provided a new perspective on the clinical application of CZTL, and it contribute to explore novel therapeutic drug against microcirculatory dysfunction.

## 1 Introduction

Ischemic stroke, also called cerebral infarction or cerebral embolism, is one of the most common cerebrovascular diseases. According to the World Health Organization, incidences of stroke have been increased by 70.0% in the past 30 years and approximately 80% stroke cases are caused by ischemic strokes (Feigin et al., 2022). Ischemic stroke is characterized by high rates of mortality and disability and poor prognosis (Montellano et al., 2021). The key of ischemic strokes treatment is to restore the cerebral blood flow perfusion in the ischemic area (K. Yang et al., 2021). However, microcirculatory dysfunction is a major cause of poor prognosis for patients with ischemic stroke (Xu et al., 2022). Microcirculation-targeted interventions to improve outcomes after ischemic stroke need to be sought.

The microcirculation is the terminal vascular network of the systemic circulation consisting of micro-vessels with diameters less than 100 µm (den Uil et al., 2008). Microcirculation plays a vital role in oxygen delivery and is responsible for the transport of all blood-borne hormones and nutrients to the tissue cells (Gutterman et al., 2016). Microcirculatory damage can be caused by ischemia, reperfusion, inflammation, and hypoxia (Widgerow, 2014); then the imbalance between vasodilating and vasoconstricting substances, increased vascular permeability, altered blood cell rheology and coagulation defects begin to appear (Colbert and Schmidt, 2016). In turn, microcirculatory dysfunction increases poor outcomes and mortality in patients with stroke (Mejía-Rentería et al., 2019). Clinicians assess the blood flow of microcirculation to guide management of patients. Direct techniques were developed to visualize the smallest vessels, such as micro-videoscope techniques, video capillaroscopy, laser speckle contrast imaging (LSCI) and side-stream darkfield imaging (Jansen et al., 2018). Plenty of trials have shown vasodilator therapy and antithrombotic therapy are well-established methods to ameliorate microcirculatory dysfunction (den Uil et al., 2009; Matskeplishvili et al., 2021). In clinical practice, the majority of the commonly used medications has been shown to be ineffective, and how best to treat microcirculatory dysfunction is still unclear (Krishnan et al., 2021).

Traditional Chinese medicine is increasingly used to relieve the symptom of stroke, because of its low-toxicity, few-side effects and low-cost (Hao et al., 2017). *Chuanzhitongluo* capsule (CZTL) is comprised of four blood-activating and stasis-removing drugs: *Whitmania pigra Whitman* (shuizhi), *Ligusticum chuanxiong Hort.* (chuanxiong), *Salvia miltiorrhiza bunge* (danshen)*, and Astragalus membranaceus* (huangqi). It has the function of activating blood circulation to dissipate blood stasis, and reinforcing *Qi* to dredge collaterals. Clinically, CZTL is accessible for using in restorative treatments of ischemic stroke and is conducive to a good prognosis (Jingwen, 2020; Jiankang and Jing, 2021). Microcirculatory dysfunction is the primary obstacles to achieving a good prognosis in ischemic stroke patients (Xu et al., 2022). Thus, we infer that the significant clinical efficacy of CZTL may dependent on its effect on microcirculatory dysfunction. CZTL is previously reported to reduce the platelet maximum aggregation rate, fibrinogen and D-dimer content in ischemic stroke patients (Jingwen, 2020; Shijun et al., 2021). It has been shown that mechanisms of action of CZTL include resistance to neuroinflammation and oxidative stress and inhibition cell apoptosis (Haiyan et al., 2021; Yanfang et al., 2021; Yaping et al., 2021). However, efficacy and mechanism of CZTL on microcirculatory dysfunction remain elusive.

In the present study, we established an acute microcirculatory dysfunction (AMD) rat model using an ice bath and epinephrine hydrochloride. The effects of CZTL on microcirculatory dysfunction were evaluated by measuring blood flow, hemodynamic and coagulation function, vasomotor factor and microcirculation function. Untargeted metabolomic techniques revealed the molecular mechanism of CZTL against microcirculatory dysfunction. This study provided a foundation for further exploration and application of CZTL, and suggested a unique therapeutic strategy for microcirculatory dysfunction.

## 2 Materials and methods

### 2.1 Chemicals and reagents

Acetylsalicylic acid (ASA) (Dalian MeilunBio Co., Ltd.) was prepared into 0.01 g/ml suspension with water. *Chuanzhitongluo* capsule (Lunan Hope Pharmaceutical Co., Ltd., Linyi City, Shandong Province, China; the batch number: 16200051) was prepared into 1 g/ml suspension with water. Epinephrine hydrochloride injection (EHI) (Grandpharma China Co., Ltd., Wuhan City, Hubei Province, China) was provided by Department of pharmacy, Guangdong Provincial Hospital of Chinese Medicine. Urethane were purchased from Aladdin. Activated partial thromboplastin time (APTT) reagent, CaCl_2_ solution, prothrombin time (PT) reagent, thrombin time (TT) reagent, fibrinogen (FIB) reagent and buffer solution were purchased from Chengdu Aikesilun Medical Technology Co., Ltd. (Chengdu, China). Chromatography-grade formic acid and methanol were obtained from Thermo Fisher Scientific, United States.

### 2.2 Animals and treatments

All animal protocols were approved by the *Ethical Committee of Guangdong Provincial Hospital of Chinese Medicine* (approval number: 2021011). Male SD rats aged 8–10 weeks (220 ± 20 g) were obtained from Guangdong Medical Laboratory Animal Center (license key: 44007200101062), and they were bred and maintained at the Guangdong Provincial Hospital of Chinese Medicine (license key: 00298102). The room temperature was maintained at 25°C with a 12 h: 12 h light-dark cycle. The mice were randomly divided into six groups (12 mice/group): control group (CON), model group (MOD), positive control group (POS), CZTL low-dose administration group (CZTL-L), CZTL medium-dose administration group (CZTL-M) and CZTL high-dose administration group (CZTL-H). During 7 days before AMD modeling, the POS group was administered ASA (0.1 g/kg) by gavage; CZTL administration groups were administered CZTL (a low dose of 0.16 g/kg, a medium dose of 0.32 g/kg and a high dose of 0.64 g/kg) by gavage; and the other groups were administered water (1 ml/100 g). ASA and CZTL was dissolved in water, and dosages were calculated based on the DuBois formula (Nair et al., 2018).

### 2.3 Rats model of acute microcirculation dysfunction

The rats from MOD, POS and CZTL groups received subcutaneous injection administration of 0.8 mg/kg EHI. After 2 h, the rats were soaked in ice-cold water for 4 min, and then re-injected with EHI after a further 2 h. Control rats received a similar volume of physiological saline at the same time points. The rats were fasted overnight and sacrificed after 12 h.

### 2.4 Sample collection

After 12 h of modeling, all rats were anaesthetized with urethane (1.5 g/kg, intraperitoneal). Rats blood was collected from abdominal aorta. 2 ml blood was collected into vacuum tubes with sodium citrate (1:9) for coagulation function detection, 4 ml blood was collected in vacuum tubes with heparin sodium for hemorheology detection and the remaining blood was collected in vacuum tubes for serum metabolomics analysis. Blood was centrifuged at 3500 rpm at 4°C for 10 min after collection. The separated serum samples were put into −80°C before further use.

### 2.5 Efficacy evaluations

#### 2.5.1 Laser speckle contrast imaging

Laser speckle contrast imaging (LSCI) was performed using PeriCam PSI System (version: PeriCam PSI NR). A 785 nm laser was used for blood perfusion measurements, and the speckle pattern in the illuminated area was monitored using a 2448 × 2048 pixel CCD camera that took images at a speed of up to 120 frames per second. To facilitate the positioning of the imager relative to the subject, a visible red laser (650 nm) was used to indicate the maximum measurement area at a certain measurement distance. Rats were placed on a flat table to keep them in the correct position and to ensure that the working distance between the laser head and the surface of foot (or ear or tail) was 100 mm. A separate color camera was used for documentation, and the image frame was chosen to be 40 mm (width) × 30 mm (height). A sampling frequency of 16 Hz was chosen, and the average was calculated from five images which left an effective frame rate of one image/s. Pseudo-color images with perfusion scaled from blue (low perfusion) to red (high perfusion) were obtained. On the basis of the real-time graphs provided by the PimSoft version 1.5 software (Perimed AB, Stockholm, Swedish), ROIs (regions of interest) were measured in feet, ears and tails of rats. The semiquantitative perfusion unit as well as the area were averaged over the sampling period. The average perfusion was calculated by PimSoft version 1.5 software.

#### 2.5.2 Coagulation function detection

The blood in sodium citrate vacuum tube was centrifuged at 3500 rpm for 10 min, and tubes were set into sample rack of EC6800 automatic coagulation analyzer (Chengdu Aikesilun Medical Technology Co., Ltd., Chengdu, China). The automatic coagulation analyzer automatically absorbed plasma 50 μl into the sample cup and kept it warm at 37°C for 1 min, and APTT reagent 50 μl was added and mixed. The mixture was kept warm for 1 min and 0.025 mol/L CaCl_2_ solution 50 μl was added. Then the coagulation time was recorded. Another 50 μl plasma was absorbed into the sample cup, kept at 37°C for 3 min, and mixed with 100 μL PT reagent, and the coagulation time was recorded. 100 μl plasma was collected, preheated at 37°C and mixed with 100 μL TT reagent, and the coagulation time was recorded. At last, 200 μl of plasma was diluted with buffer solution (1:10) and kept it warm at 37°C for 3 min, and 100 μl of thrombin reagent was added to the tube to measure the content of FIB.

#### 2.5.3 Hemorheology detection

The heparin sodium anticoagulant tubes were put into sample tray of MEN-C100A blood rheometer (Jinan Meiyilin Electronic Instrument Co., Ltd., Jinan, China), and high, medium and low shear values of blood viscosity were detected. Then the heparin sodium anticoagulant tubes were centrifuged at 3500 rpm for 10 min, and the plasma viscosity was measured by MEN-C100A blood rheometer.

#### 2.5.4 ELISA assays

ELISA kits of NOS (ml059067-2), vWF (ml003160-2), VE-cadherin (ml782930-2) and IL-6 (ml102828-2) were obtained from Shanghai Enzyme-linked Biotechnology Co., Ltd. (Shanghai, China). The detection was approached according to the instructions of kits on rat plasma.

### 2.6 Untargeted metabolomics analysis

#### 2.6.1 Sample preparation

Frozen serum samples were thawed and dissolved at 4°C. The serum (200 μl) was transferred to clean Eppendorf tubes. Threefold volume of methanol was added to each tube. The mixture was then vortexed for 1 min and kept undisturbed at 4°C for 30 min, followed by centrifugation at 14000 rpm for 10 min. The supernatant was transferred to a clean Eppendorf tube, filtered for metabolomics analysis. Quality control (QC) samples were prepared by mixing the same amount of serum from each sample and using the same procedure as test samples to extract metabolites. Methanol solvent was used as the blank sample.

#### 2.6.2 Serum metabolomics analysis

Metabolite separation was performed using a Waters acquity ultra high performance liquid chromatography (UPLC) system (Waters Corp., Milford, Massachusetts, United States) coupled with a quadrupole time-of-flight mass spectrometer (TripleTOF^®^ 5600 + System; AB SCIEX, Framingham, Massachusetts, United States). Liquid-chromatographic separation for processed serum samples was achieved on an Acquity UPLC BEH C18 column (2.1 mm × 100 mm, 1.7 μm) maintained at 35°C. The mobile phase consisted of water with 0.1% formic acid (mobile phase A) and methanol (mobile phase B). The gradient program began with 2% B at 0–2 min, 2%–20% B at 2–3, 20%–40% B at 3–9, 40% B at 9–18, 40%–72% B at 18–20, 72%–75% B at 20–25, 75%–77% B at 25–26, 77%–85% B at 26–36, 85%–94% B at 36–38, 94%–95% B at 38–50, 95%–98% B at 50–52, and 98% B at 52–56 and then returned to the initial conditions with 8 min for equilibration. The flow rate was 0.3 ml/min. The sample injection volumes were 2 μl.

Mass spectrometry was performed with an electrospray ionization ion source in the positive (ESI+) and negative (ESI−) ion modes. The flow rate of ion source gas 1, ion source gas two and the curtain gas were set as 50 psi, 50 psi, and 35 psi respectively. The capillary voltage was set to 5500 V/-4500 V at ion source temperature of 500°C. The declustering potential voltage was 80V/-100 V. TOF-MS survey scan (100–2000 Da) followed by 6 MS/MS scans (50–1500 Da) with accumulation time of 0.25 and 0.1 s respectively. The collision energy was set to −10 V and set to −40 V with a spread of ± 20 V for MS/MS. For product ion, the ion release delay was 67 and the ion cluster width was 25. Both the negative and positive ion modes were applied with dynamic background subtraction.

### 2.7 Method validation

The precision, repeatability and stability of the analytical procedure were validated using QC sample. Calibration solution and blank samples analyzed at every five injection intervals, and QC samples were inserted into every ten samples regularly.

### 2.8 Data processing

LC-MS raw data were deconvolved using Progenesis QI (Waters Corp., Milford, Massachusetts, United States). Peak picking, alignment, and area normalisation were carried out using one of the QC data files as the reference. Significant features extracted from raw data were aligned to significant features in the reference sample, using a RT window ± 0.2 min and mass tolerance ± 5 ppm filters. Features were annotated using accurate mass match and tandem MS data with the human metabolome database (HMDB). Mass tolerances of 5 and 5 ppm were applied for precursor and fragment ions, respectively. Compounds with a fragmentation score <20 were not annotated. Progenesis QI score, fragmentation score, and isotope similarity were reported for all annotations based on a combination of accurate mass and fragmentation data, seen in Supplementary Table S1 concluding 3461 metabolite features in negative ion mode and 2426 metabolite features in positive ion mode.

### 2.9 Statistical analysis

The integrated data matrix was imported into the SIMCA-P + (version 13.0) software package (Umetrics, Umeå, Sweden), and the principal component analysis (PCA) and orthogonal partial least-squares discriminant analysis (OPLS-DA) were used to distinguish the overall difference in metabolic profile among groups. The variable important in projection (VIP) value was calculated and obtained based on OPLS-DA. Metabolites with VIP >1 were further subjected to univariate statistical analysis to measure the significance of each metabolite (seen in Supplementary Table S2).

The data was expressed as the mean ± standard deviation (SD). The univariate statistical analysis were performed using IBM SPSS statistics 18.0 software (SPSS Inc., Chicago, Illinois, United States). Firstly, the Levene’s test was used for homogeneity of variances. And then, one-way analysis of variance (ANOVA) with Dunnett t post hoc test was used for homogeneous variances, while ANOVA with Tamhane post hoc test was used for non-homogeneous variances. *p* value less than 0.05 or 0.01 was considered statistically significant. The condition of VIP >1 and *p* < 0.05 was used to screen the differential expressed metabolites.

### 2.10 Pathway analysis

Pathway analysis of differential expressed metabolites was performed using MetaboAnalyst 5.0 (https://www.metaboanalyst.ca). Metabolites were mapped onto *Homo sapiens* Kyoto Encyclopaedia of Genes and Genomes (KEGG) metabolic network. *p* value of pathway were calculated by hypergeometric test and pathway impact were obtained from relative-betweeness centrality of topology analysis.

## 3 Results

### 3.1 Efficacy of *Chuanzhitongluo* capsule in acute microcirculatory dysfunction rats

#### 3.1.1 *Chuanzhitongluo* capsule increased local blood flow in the acute microcirculatory dysfunction rats

**FIGURE 1:**
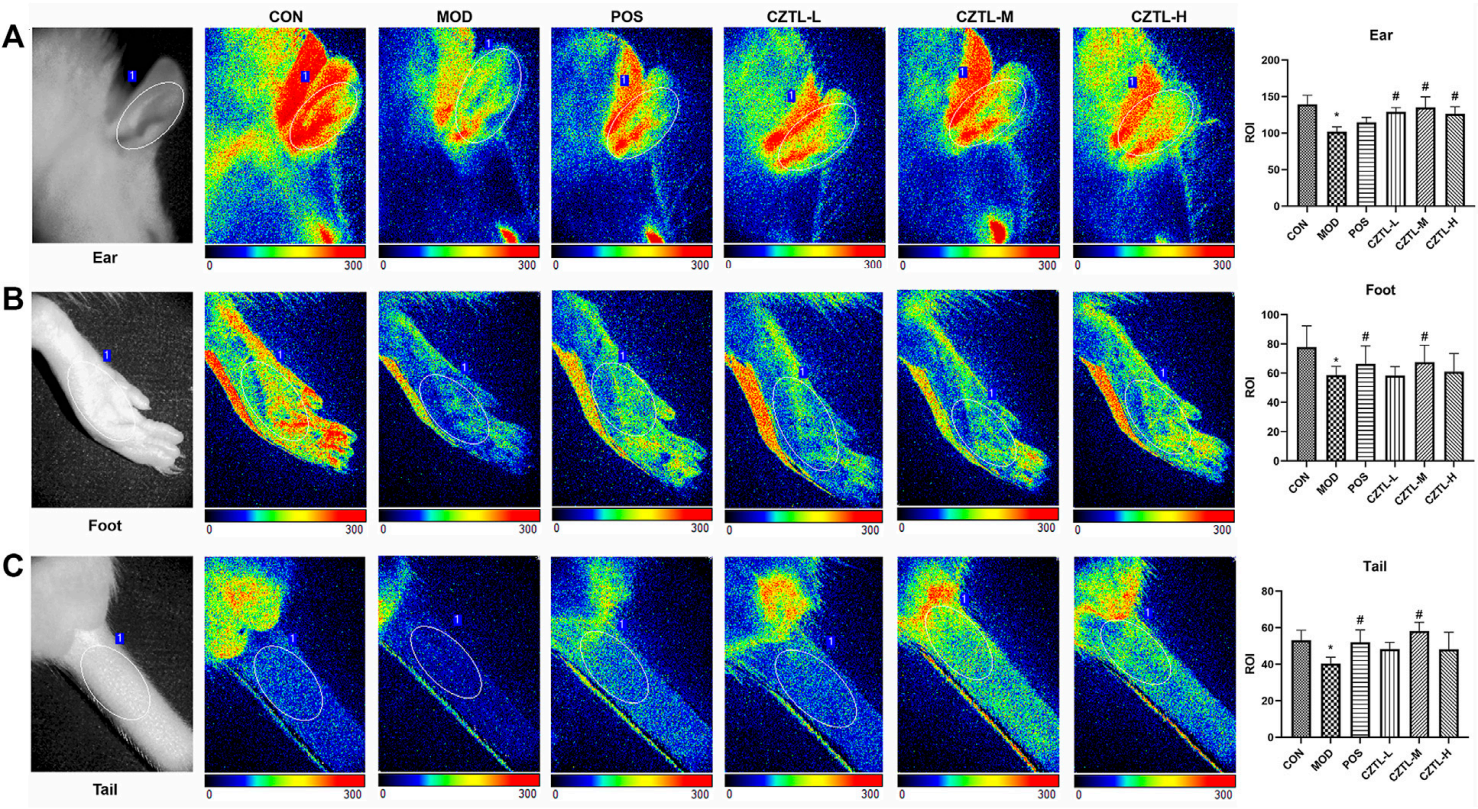
The laser speckle contrast imaging image of rats ears **(A)**, feet **(B)** and tails **(C)**. CON, control group (*n* = 7); MOD, model of acute microcirculation dysfunction group (*n* = 6); POS, positive control group (*n* = 5); CZTL-L, *Chuanzhitongluo* capsule low-dose administration group (*n* = 7); CZTL-M, CZTL medium-dose administration group (*n* = 6); CZTL-H, CZTL high-dose administration group (*n* = 8). Statistical significance indicates as asterisk (*) when comparing CON group with MOD group, and as hashtag (#) when POS, CZTL-L/M/H group with MOD group. * presents *p* < 0.05, # presents *p* < 0.05.

To make ensure that the interval time between modeling and blood collection was the same, some rats had no time to carry out laser speckle contrast imaging. Finally, rats from CON group (*n* = 7), MOD group (*n* = 6), POS group (*n* = 5), CZTL-L group (*n* = 7), CZTL-M group (*n* = 6), and CZTL-H group (*n* = 8) were able to obtain images of laser speckle contrast imaging. A region of interest (marked with a white ellipse in Figures 1A–C) was selected for maximizing the immutability of the analyzed area. The speckle contrast values for each frame were averaged over space, and the statistical processing results of microvascular blood flow changes in ears, feet and tails of different groups were displayed in the form of histogram.

LSCI analyses showed decreases of microvascular blood flow in ears of AMD rats compared to healthy rats. In addition, compared with the AMD rats, pre-administration of ASA and CZTL significantly increased blood flow of ears. As in the case of ears, more microvascular blood flow were observed in feet and tails of drug administration groups.

#### 3.1.2 *Chuanzhitongluo* capsule restored coagulation function in the acute microcirculatory dysfunction rats

**FIGURE 2:**
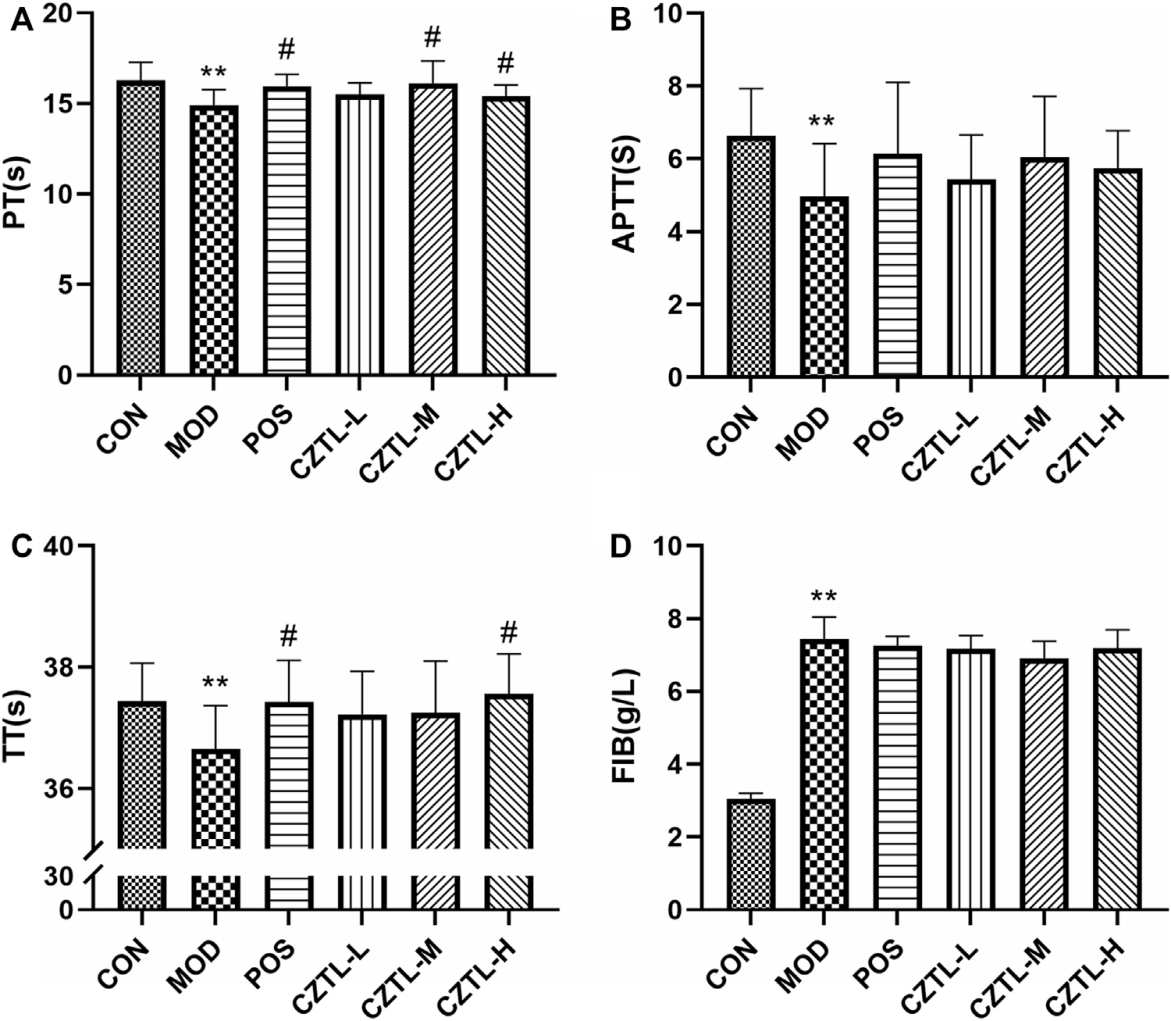
The results of coagulation function test. **(A)**, prothrombin time (PT); **(B)**, activated partial thromboplastin time (APTT); **(C)**, thrombin time (TT); **(D)**, fibrinogen (FIB). CON, control group (*n* = 9); MOD, model of acute microcirculation dysfunction group (*n* = 8); POS, positive control group (*n* = 8); CZTL-L, *Chuanzhitongluo* capsule low-dose administration group (*n* = 9); CZTL-M, CZTL medium-dose administration group (*n* = 9); CZTL-H, CZTL high-dose administration group (*n* = 7). Statistical significance indicates as asterisk (*) when comparing CON group with MOD group, and as hashtag (#) when POS, CZTL-L/M/H group with MOD group. * presents *p* < 0.05, # presents *p* < 0.05.

The effect of CZTL on blood coagulation function was measured by assessment of TT, PT, APTT and FIB contents in CON group (*n* = 9), MOD group (*n* = 8), POS group (*n* = 8), CZTL-L group (*n* = 9), CZTL-M group (*n* = 9), and CZTL-H group (*n* = 7). As illustrated in Figure 2, the TT, PT and APTT value were significantly decreased (*p* < 0.01), and the FIB level (*p* < 0.01) was significantly elevated in AMD rats in comparison to healthy rats. The CZTL and ASA administration both prolonged time of TT, PT, and APTT as well as down-regulated the FIB content, and changes of PT and TT were statistically significant (PT: POS vs. MOD *p* < 0.05, CZTL-M vs. MOD *p* < 0.05, CZTL-H vs. MOD *p* < 0.05; TT: POS vs. MOD *p* < 0.05, CZTL-H vs. MOD *p* < 0.05). Results showed that CZTL medium-dose and high-dose administration showed stronger effects for coagulation function than CZTL low-dose administration.

#### 3.1.3 *Chuanzhitongluo* capsule improved hemorheology parameters in the acute microcirculatory dysfunction rats

**FIGURE 3:**
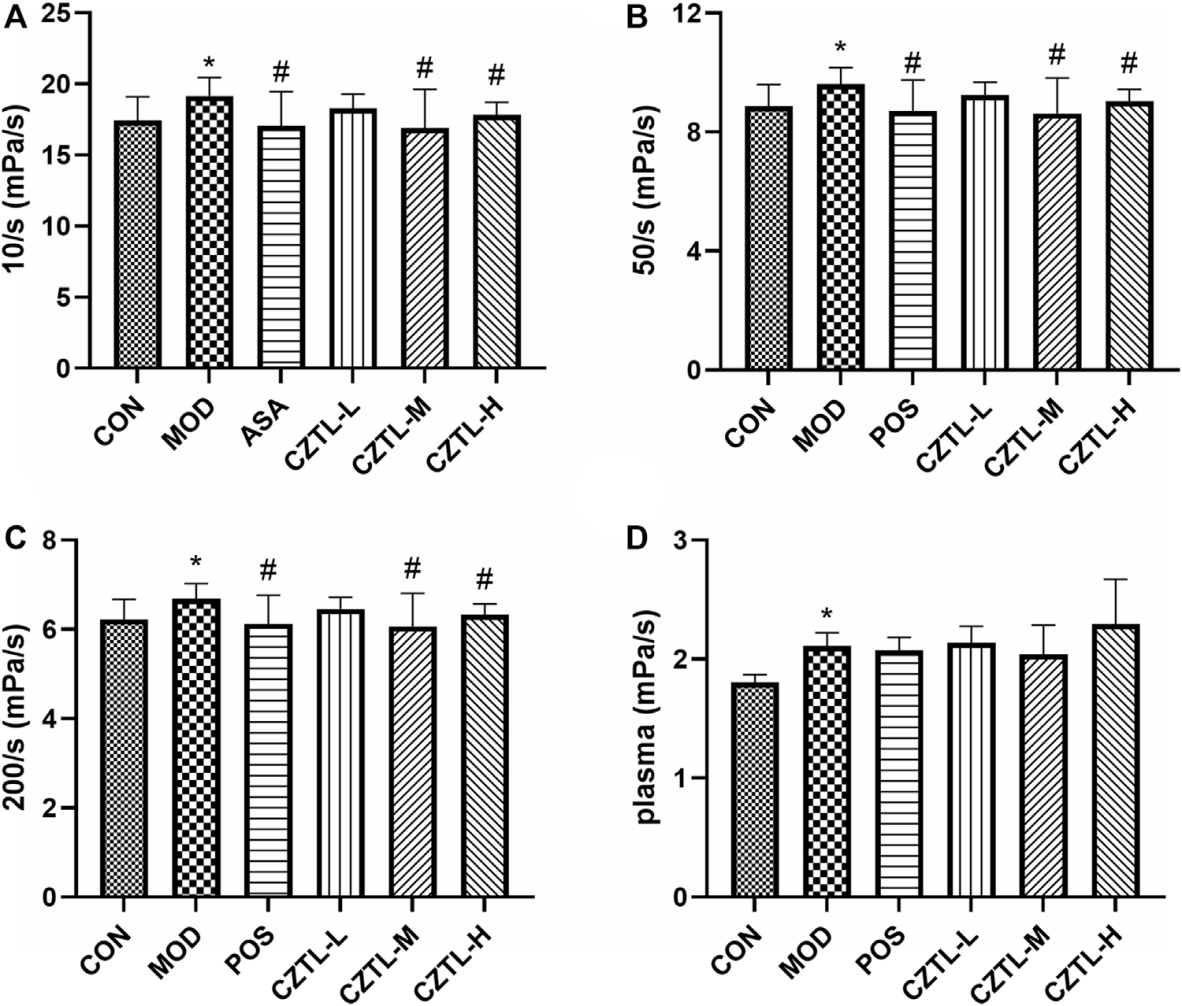
The results of hemorheology parameters detection. **(A)**, whole blood viscosity (WBV) at low shear rate; **(B)**, WBV at medium shear; **(C)**, WBV at high shear; **(D)**, plasma viscosity. CON, control group (*n* = 9); MOD, model of acute microcirculation dysfunction group (*n* = 8); POS, positive control group (*n* = 8); CZTL-L, *Chuanzhitongluo* capsule low-dose administration group (*n* = 9); CZTL-M, CZTL medium-dose administration group (*n* = 9); CZTL-H, CZTL high-dose administration group (*n* = 7). Statistical significance indicates as asterisk (*) when comparing CON group with MOD group, and as hashtag (#) when POS, CZTL-L/M/H group with MOD group. * presents *p* < 0.05, # presents *p* < 0.05.

Rats from CON group (*n* = 9), MOD group (*n* = 8), POS group (*n* = 8), CZTL-L group (*n* = 9), CZTL-M group (*n* = 9), and CZTL-H group (*n* = 7) were included for the hemorheological detection. The whole blood viscosity (WBV) at low shear rate, WBV at medium shear rate, WBV at high shear rate and plasma viscosity (PV) were evaluated. The WBV and PV significantly increased in the MOD group (*p* < 0.01, Figure 3), which indicated the rat model of AMD were successfully established. In comparison to the MOD group, the WBV at low/medium/high shear rate remarkably decreased in POS and CZTL (CZTL-M and CZTL-H) groups (*p* < 0.05). CZTL low-dose administration also caused the decrease of WBV and PV with no statistically significant (*p* > 0.05).

#### 3.1.4 *Chuanzhitongluo* capsule affected biochemical indexes in the acute microcirculatory dysfunction rats

**FIGURE 4:**
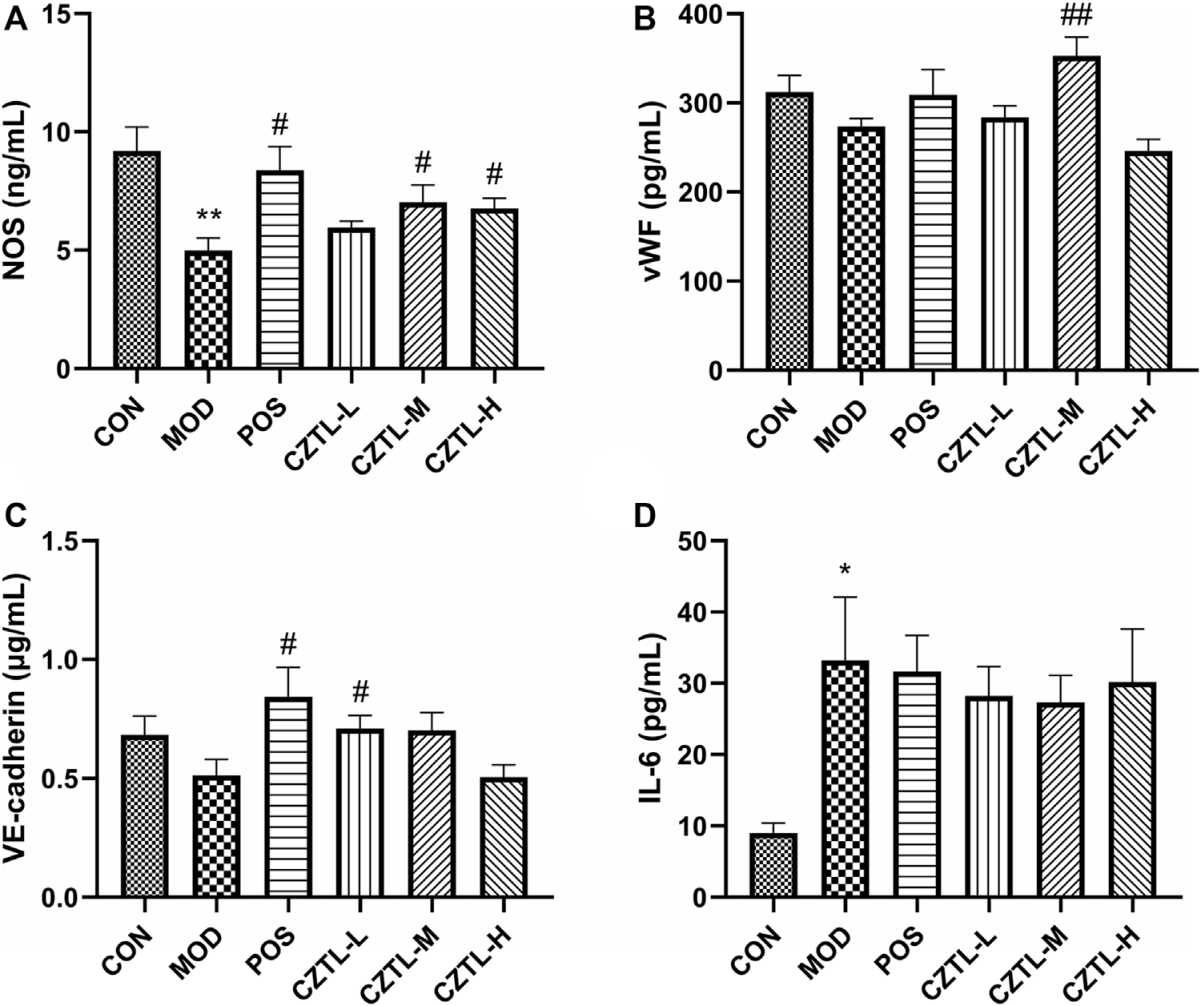
The results of biochemical indexes assay. **(A)**, nitric oxide synthase (NOS); **(B)**, von Willebrand factor (vWF); **(C)**, vascular endothelial (VE)-cadherin; **(D)**, interleukin-6 (IL-6). CON, control group (*n* = 7); MOD, model of acute microcirculation dysfunction group (*n* = 6); POS, positive control group (*n* = 7); CZTL-L, *Chuanzhitongluo* capsule low-dose administration group (*n* = 8); CZTL-M, CZTL medium-dose administration group (*n* = 8); CZTL-H, CZTL high-dose administration group (*n* = 7). Statistical significance indicates as asterisk (*) when comparing CON group with MOD group, and as hashtag (#) when POS, CZTL-L/M/H group with MOD group. * presents *p* < 0.05, # presents *p* < 0.05.

Rats plasma from CON group (*n* = 7), MOD group (*n* = 6), POS group (*n* = 7), CZTL-L group (*n* = 8), CZTL-M group (*n* = 8), and CZTL-H group (*n* = 7) were used for ELISA assays. Nitric oxide produced by nitric oxide synthase (NOS) was a key factor in vasodilation. We found that NOS level was significantly increased in ASA or CZTL groups (*p* < 0.05), while it was decreased in MOD group (*p* < 0.01) (Figure 4A). The vascular endothelial (VE)-cadherin regulating endothelial intercellular permeability were decreased in AMD rats, and treatment of ASA or CZTL (low-dose and medium-dose) could significantly increase the VE-cadherin to normal levels (*p* < 0.05) (Figure 4C). In addition, compared with the healthy rats, the AMD rats showed a decline trend in von Willebrand factor (vWF) content (*p* < 0.01), which was crucial to repair vascular damage (Figure 4B). Administration of ASA or CZTL (low-dose and medium-dose) counterbalanced the decreased vWF level in AMD rats (*p* < 0.01). Finally, we also examined the effects of CZTL on inflammatory responses in AMD rats. As shown in Figure 4D, AMD rats exhibited extremely high level of interleukin-6 (IL-6) (*p* < 0.01), which was reduced in ASA or CZTL groups; CZTL administration partially reversed the inflammatory responses in AMD rats.

### 3.2 Mechanism insight of *Chuanzhitongluo* capsule against microcirculatory dysfunction

#### 3.2.1 *Chuanzhitongluo* capsule influenced metabolic state in the acute microcirculatory dysfunction rats

**FIGURE 5:**
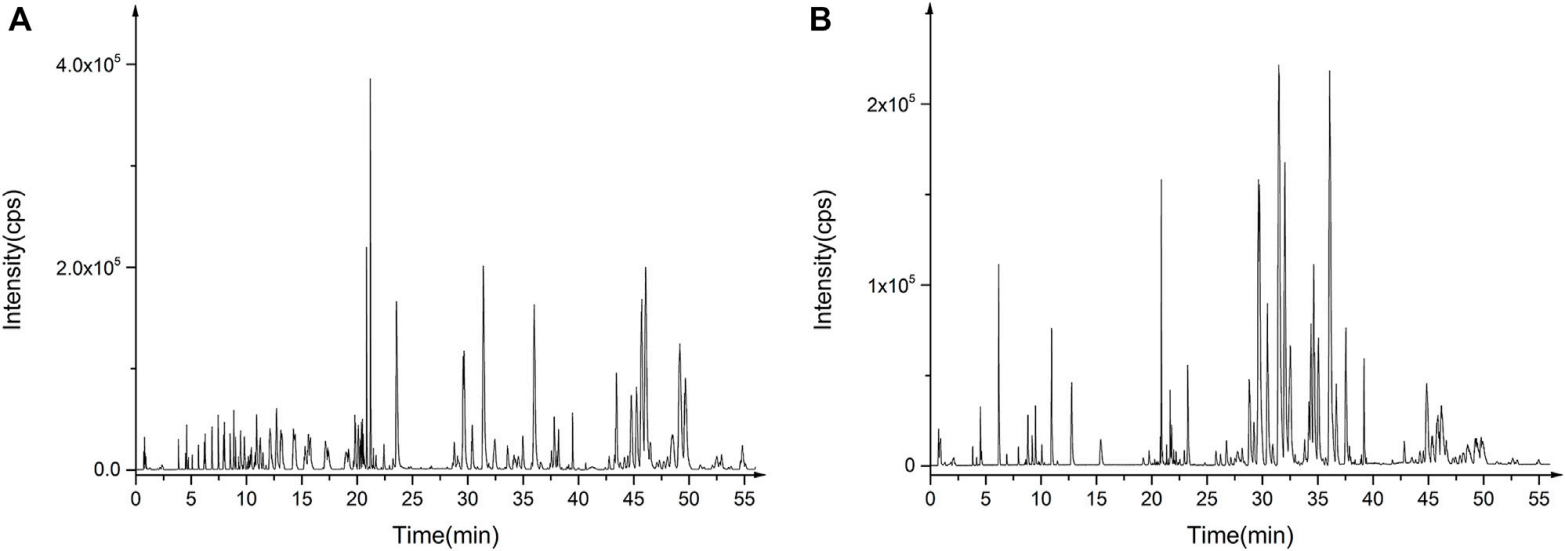
The base peak chromatogram of quality control samples in positive ion mode **(A)** and in negative ion mode **(B)**.

**FIGURE 6:**
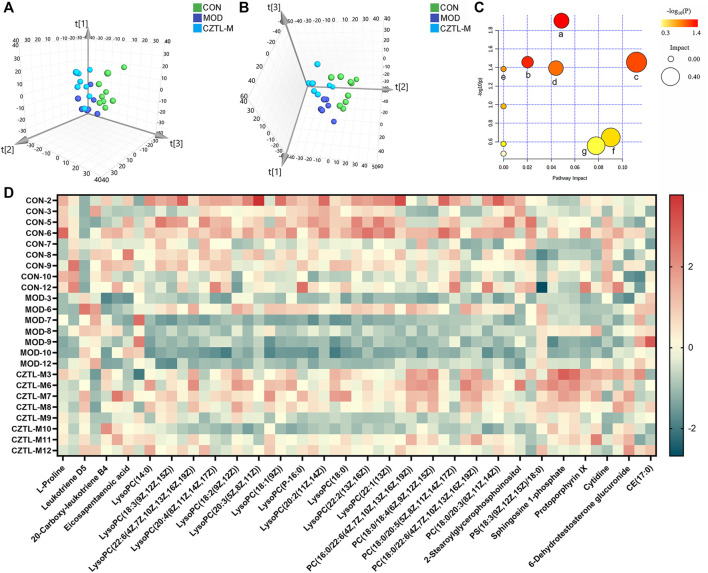
The results of metabolomics analysis. **(A)**, the score plot of principal component analysis in positive ion mode; **(B)**, the score plot of principal component analysis in positive ion mode in negative ion mode; **(C)**, the results of pathway analysis. a represents sphingolipid metabolism, b represents arachidonic acid metabolism, c represents glycerophospholipid metabolism, d represents pyrimidine metabolism, e represents linoleic acid metabolism, f represents porphyrin and chlorophyll metabolism, g represents arginine and proline metabolism; **(D)**, the heat map of 55 differentially expressed metabolites. CON, control group (*n* = 9); MOD, model of acute microcirculation dysfunction group (*n* = 7); CZTL-M, *Chuanzhitongluo* capsule medium-dose administration group (*n* = 8).

To explore the mechanism of CZTL against microcirculatory dysfunction, we undertook an untargeted metabolomics analysis of rats serum from CON group (*n* = 9), MOD group (*n* = 7), and CZTL-M group (*n* = 8). In view of the similarity between the efficacy of medium-dose and high-dose of CZTL and the consideration of drug safety, we selected the CZTL-M group for the subsequent analysis. The UPLC-Q/TOF MS was used to obtain metabolic spectrums of serum samples (Figure 5). The regression model of PCA was constructed to predict differences among CON group, MOD group and CZTL-M group. From PCA model score plots (Figures 6A,B), positive and negative ion points had a significantly separated trend between CON group and MOD group, indicating metabolites in AMD rats serum were different from that in healthy rats. Moreover, the metabolic profile of AMD and CZTL-M showed a clear tendency for separation. Our result showed that microcirculatory dysfunction caused metabolic disorders and CZTL administration also alter the metabolic status of AMD rats.

#### 3.2.2 *Chuanzhitongluo* capsule changed metabolites expression in the acute microcirculatory dysfunction rats

In order to identify the characteristic metabolites responsible for the separations among the CON group, MOD group and CZTL-M group, OPLS-DA models visualized the metabolic differences were established. VIP of OPLS-DA model embodied the impact strength and explanatory power of metabolite expression patterns on the classification and discrimination of each group sample. According to the condition of VIP > 1.0 and *p* < 0.05, there were 62 differentially expressed metabolites in serum between the CON group and MOD group, with 37 downregulated and 25 upregulated metabolites in AMD rats. Comparing with AMD rats, CZTL administration led to 41 metabolites upregulated and two metabolites downregulated (Figure 6C). Taken together, 55 metabolites showed significant callback trends in CZTL-M group (Table 1). They were glycerophospholipids, nucleosides, eicosanoids, fatty acids, phosphosphingolipids, porphyrins, and amino acids and others.

**TABLE 1:** Detailed information of 55 differentially expressed metabolites.

No.	Adduct Type	m/z	RT (min)	Compound ID	Description	Class	Intensity in MOD (*n* = 7)	Intensity in CON (*n* = 9)	Intensity in CZTL-M (*n* = 8)
**1**	[M + H]^+^	266.0738	0.86	HMDB0000162	L-Proline	Amino acids, peptides, and analogues	13.2 ± 2.26	21.51 ± 6.18	18.17 ± 2.59
**2**	[M + H]^+^	367.1208	0.86	HMDB0000089	Cytidine	Pyrimidine nucleosides	7.53 ± 1.12	10.41 ± 1.48	7.98 ± 1.74
**3**	[M + H]^+^	242.9831	1.24	HMDB0060689	Phosphoramide mustard	Nitrogen mustard compounds	5.73 ± 1.24	6.67 ± 1.43	8.66 ± 1.81
**4**	[M + H]^+^	258.1075	1.66	HMDB0000982	5-Methylcytidine	Pyrimidine nucleosides	3.55 ± 0.33	4.24 ± 0.62	4.27 ± 0.63
**5**	[M + H]^+^	384.1137	4.27	HMDB0000912	Succinyladenosine	Purine nucleosides	2.91 ± 1.26	3.14 ± 0.88	4.7 ± 1.31
**6**	[M-H]^-^	565.1967	5.14	HMDB0012994	Leukotriene D5	Eicosanoids	27.39 ± 7.85	11.35 ± 2.77	25.08 ± 5.39
**7**	[M-H]^-^	119.0500	7.80	HMDB0062775	4-vinylphenol Sulfate	Arylsulfates	23.03 ± 10.92	55.69 ± 19.24	26.61 ± 7.54
**8**	[M-H]^-^	858.5517	20.03	HMDB0012382	PS(18:0/20:3 (8Z,11Z,14Z))	Glycerophosphoserines	3.03 ± 0.66	4.7 ± 1.73	3.22 ± 1.22
**9**	[M-H]^-^	335.2218	21.16	HMDB0001085	Leukotriene B4	Eicosanoids	13.12 ± 2.55	10.8 ± 2.65	8.03 ± 2.4
**10**	[M-H]^-^	512.2981	27.14	HMDB0010379	LysoPC(14:0)	Glycerophosphocholines	604.26 ± 156.31	915.82 ± 260.8	990.86 ± 262.06
**11**	[M + H]^+^	518.3224	27.72	HMDB0010382	LysoPC(16:0)	Glycerophosphocholines	64.07 ± v18.27	108.64 ± 26.71	89.49 ± 11.35
**12**	[M-H]^-^	562.3132	27.76	HMDB0010388	LysoPC(18:3 (9Z,12Z,15Z))	Glycerophosphocholines	333.43 ± 82.48	453.26 ± 106.15	452.51 ± 46.2
**13**	[M-H]^-^	480.1648	28.03	HMDB0000277	Sphingosine 1-phosphate	Phosphosphingolipids	946.21 ± 295.82	1035.72 ± 224.94	1832.06 ± 586.36
**14**	[M + H]^+^	494.3237	28.13	HMDB0010383	LysoPC(16:1 (9Z))	Glycerophosphocholines	525.68 ± 199.47	1251.79 ± 424.36	804.58 ± 225.34
**15**	[M-H]^-^	335.2201	28.39	HMDB0014347	Succinylcholine	Quaternary ammonium salts	9.61 ± 1.83	6.11 ± 2.36	7.19 ± 2.82
**16**	[M-H]^-^	588.3296	28.86	HMDB0010404	LysoPC(22:6 (4Z,7Z,10Z,13Z,16Z,19Z))	Glycerophosphocholines	1370.07 ± 467.68	1714.79 ± 282.19	2004.9 ± 479.77
**17**	[M + H]^+^	482.3229	29.18	HMDB0010381	LysoPC(15:0)	Glycerophosphocholines	356.36 ± 96.13	657.43 ± 138.68	465.38 ± 138.02
**18**	[M-H]^-^	380.2565	29.41	HMDB0001383	Sphinganine 1-phosphate	Phosphosphingolipids	180.78 ± 73.56	182.14 ± 47.81	416.84 ± 134.31
**19**	[M + H]^+^	544.3397	29.59	HMDB0010396	LysoPC(20:4 (8Z,11Z,14Z,17Z))	Glycerophosphocholines	17355.96 ± 3141.48	27142.67 ± 3767.24	20578.88 ± 2578.97
**20**	[M-H]^-^	588.3300	29.64	HMDB0010395	LysoPC(20:4 (5Z,8Z,11Z,14Z))	Glycerophosphocholines	31859.89 ± 3568.08	43718.46 ± 3782.86	37600.31 ± 4605.97
**21**	[M-H]^-^	564.3300	29.74	HMDB0010386	LysoPC(18:2 (9Z,12Z))	Glycerophosphocholines	34455.31 ± 5984.84	35171.02 ± 5553.49	41544.27 ± 5640.4
**22**	[M-H]^-^	614.3448	30.09	HMDB0010402	LysoPC(22:5 (4Z,7Z,10Z,13Z,16Z))	Glycerophosphocholines	283.23 ± 121.98	438.81 ± 138.94	389.85 ± 120.57
**23**	[M + H]^+^	580.2903	30.42	HMDB0000241	Protoporphyrin IX	Porphyrins	52.8 ± 13.36	83.29 ± 27.23	83.6 ± 17.09
**24**	[M-H]^-^	590.3452	31.40	HMDB0010393	LysoPC(20:3 (5Z,8Z,11Z))	Glycerophosphocholines	538.68 ± 102.74	1147.8 ± 540.88	641.76 ± 172.82
**25**	[M-H]^-^	301.2173	31.91	HMDB0001999	Eicosapentaenoic acid	Fatty acids and conjugates	544.35 ± 184.57	792.12 ± 227.74	701.48 ± 160.08
**26**	[M + H]^+^	506.3587	31.92	HMDB0010408	LysoPC(P-18:1 (9Z))	Glycerophosphocholines	10.66 ± 5.12	14.28 ± 3.41	17.06 ± 4.18
**27**	[M-H]^-^	566.3460	32.51	HMDB0002815	LysoPC(18:1 (9Z))	Glycerophosphocholines	20142.28 ± 5278.28	28719.16 ± 6359.02	26362.47 ± 3699.74
**28**	[M-H]^-^	702.2743	32.77	HMDB0011511	LysoPE (20:0/0:0)	Glycerophosphoethanolamines	715.71 ± 179.91	1104.04 ± 289.42	943.96 ± 103.94
**29**	[M-H]^-^	616.3615	33.25	HMDB0010401	LysoPC(22:4 (7Z,10Z,13Z,16Z))	Glycerophosphocholines	463.34 ± 150.55	679.36 ± 167.44	633.33 ± 172.24
**30**	[M + H]^+^	480.3432	33.42	HMDB0010407	LysoPC(P-16:0)	Glycerophosphocholines	44.18 ± 14.19	71.23 ± 16.33	56.33 ± 14.55
**31**	[M + H]^+^	510.3551	33.75	HMDB0012108	LysoPC(17:0)	Glycerophosphocholines	905.64 ± 287.31	1438.01 ± 289.73	1213.1 ± 210.13
**32**	[M + H]^+^	548.3697	33.93	HMDB0010392	LysoPC(20:2 (11Z,14Z))	Glycerophosphocholines	175.58 ± 63.41	404.71 ± 182.36	261.16 ± 60.24
**33**	[M + H]^+^	623.4288	34.50	HMDB0013325	2-trans,4-cis-Decadienoylcarnitine	Fatty acid esters	15.91 ± 5.11	22.2 ± 6.22	24.84 ± 6.89
**34**	[M-H]^-^	291.1998	34.86	HMDB0013623	12 (13)Ep-9-KODE	Fatty acids and conjugates	46.88 ± 13.26	32.99 ± 5.23	34.02 ± 10.02
**35**	[M-H]^-^	552.3663	34.91	HMDB0013122	LysoPC(P-18:0)	Glycerophosphocholines	537.51 ± 161.74	793.06 ± 116.94	782.69 ± 193.48
**36**	[M + H]^+^	524.3710	36.01	HMDB0010384	LysoPC(18:0)	Glycerophosphocholines	35522.28 ± 7854.5	50546.35 ± 7915.08	49617.61 ± 8866.77
**37**	[M-H]^-^	594.3800	36.84	HMDB0010391	LysoPC(20:1 (11Z))	Glycerophosphocholines	1212.67 ± 400.38	2005.37 ± 481.15	1455.45 ± 236.22
**38**	[M-H]^-^	411.2056	37.55	HMDB0006059	20-Carboxy-leukotriene B4	Eicosanoids	147.57 ± 15.22	174.06 ± 15.07	171.12 ± 17.39
**39**	[M-H]^-^	620.3923	37.93	HMDB0010400	LysoPC(22:2 (13Z,16Z))	Glycerophosphocholines	23.42 ± 8.52	37.89 ± 12.13	32.74 ± 6.31
**40**	[M-H]^-^	596.3930	38.87	HMDB0010390	LysoPC(20:0)	Glycerophosphocholines	312.08 ± 114.88	614.38 ± 181.87	399.11 ± 72.8
**41**	[M-H]^-^	622.4081	39.06	HMDB0010399	LysoPC(22:1 (13Z))	Glycerophosphocholines	86.99 ± 40.07	155.56 ± 50.65	113.56±23.11
**42**	[M-H]^-^	507.2245	39.38	HMDB0010337	6-Dehydrotestosterone glucuronide	Pyrimidines and pyrimidine derivatives	5.55 ± 1.64	5.57 ± 1.95	9.57 ± 1.94
**43**	[M + H]^+^	780.5528	43.51	HMDB0008016	PC(16:1 (9Z)/20:4 (8Z,11Z,14Z,17Z))	Glycerophosphocholines	2853.88 ± 1148.07	6414 ± 3018.85	5044.88 ± 1647.11
**44**	[M-H]^-^	599.3194	45.15	HMDB0061704	2-Stearoylglycerophosphoinositol	Glycerophosphoinositols	1923.25 ± 485.94	3061.53 ± 666.53	2457 ± 809.7
**45**	[M + H]^+^	806.5699	45.25	HMDB0007991	PC(16:0/22:6 (4Z,7Z,10Z,13Z,16Z,19Z))	Glycerophosphocholines	29599.38 ± 9681.78	32714.71 ± 7074.23	44993.5 ± 9870.31
**46**	[M-H]^-^	790.5386	45.34	HMDB0007958	PC(15:0/22:6 (4Z,7Z,10Z,13Z,16Z,19Z))	Glycerophosphocholines	198.71 ± 39.23	243.58 ± 42.64	264.43 ± 44.38
**47**	[M + H]^+^	782.5705	45.71	HMDB0008042	PC(18:0/18:4 (6Z,9Z,12Z,15Z))	Glycerophosphocholines	70381.38 ± 13514.35	80288.23 ± 15965.04	100437.55 ± 20132.52
**48**	[M + H]^+^	786.5270	46.09	HMDB0012411	PS(18:3 (9Z,12Z,15Z)/18:0)	Glycerophosphoserines	410.04 ± 51.82	278.76 ± 81.33	406.66 ± 68.74
**49**	[ M + H]^+^	808.5844	46.26	HMDB0008115	PC(18:1 (9Z)/20:4 (8Z,11Z,14Z,17Z))	Glycerophosphocholines	6052.53 ± 1304.19	11054.46 ± 4142.85	8255.37 ± 1481.76
**50**	[M + H]^+^	830.5659	46.28	HMDB0008050	PC(18:0/20:5 (5Z,8Z,11Z,14Z,17Z))	Glycerophosphocholines	535.18 ± 68.47	862.25 ± 141.82	542.19 ± 55.66
**51**	[M + H]^+^	760.5866	48.49	HMDB0008067	PC(18:1 (11Z)/16:0)	Glycerophosphocholines	20305.67 ± 7080.98	20275.6 ± 5434.84	29901 ± 7099.61
**52**	[M + H]^+^	834.6017	48.55	HMDB0008057	PC(18:0/22:6 (4Z,7Z,10Z,13Z,16Z,19Z))	Glycerophosphocholines	18903.14 ± 3310.73	19529.9 ± 4173.04	25362.45 ± 5157.02
**53**	[M + H]^+^	858.5982	49.93	HMDB0008055	PC(18:0/22:5 (4Z,7Z,10Z,13Z,16Z))	Glycerophosphocholines	309.83 ± 51.9	435.86 ± 86.86	363.02 ± 71.74
**54**	[M + H]^+^	812.6180	51.01	HMDB0008047	PC(18:0/20:3 (8Z,11Z,14Z))	Glycerophosphocholines	3228.22 ± 1081.24	5436.12 ± 2178.68	4828.29 ± 1022.83
**55**	[M-H]^-^	637.5871	54.29	HMDB0060059	CE (17:0)	Steroid esters	15.61 ± 5.03	10.82 ± 2.99	11.77 ± 3.49

Notes: RT, retention time; CON, control group (*n* = 9); MOD, model of acute microcirculation dysfunction group (*n* = 7); CZTL-M, *Chuanzhitongluo* capsule medium-dose administration group (*n* = 8).

#### 3.2.3 *Chuanzhitongluo* capsule regulated metabolomic pathways in the acute microcirculatory dysfunction rats

To get more insights into the molecular mechanism of CZTL, we performed pathway analysis of 55 differentially expressed metabolites. MetaboAnalyst 5.0 was used to analyze metabolites by the MetPA (metabolomics pathway analysis) approach (Xia and Wishart, 2010). The data of metabolites was imported into Pathway Analysis to explore the weight of metabolic pathway, and 10 metabolic pathways were enriched. Particularly, seven metabolic pathways were heavily highlighted with raw *p* < 0.05 or pathway impact >0.05. They were sphingolipid metabolism, arachidonic acid metabolism, glycerophospholipid metabolism, pyrimidine metabolism, linoleic acid metabolism, porphyrin and chlorophyll metabolism, arginine and proline metabolism (Figure 6D).

## 4 Discussion

Most patients with ischemic stroke had a poor prognosis due to the microcirculatory dysfunction (Chung et al., 2019). However, available therapeutic options (ie, thrombolysis and surgical thrombectomy) were very limited so far (Miranda et al., 2016). Extensive clinical evidences supported that blood-activating and stasis-removing drugs provided excellent efficacy in improving microvascular function and clinical prognosis (Fang et al., 2022; Wang et al., 2021; M. X. Zhang et al., 2022). *Whitmania pigra Whitman* was mostly utilized in the localized venous congestion settings related to flap surgical replantation and reconstructions (Shakouri and Wollina, 2021). *Ligusticum chuanxiong Hort.* had satisfied outcome in treating cerebrovascular disease by affecting microcirculation and autophagy (Yu et al., 2020). *Salvia miltiorrhiza bunge* was a famous Chinese medicine for blood-activating and stasis-removing, and it was widely used to treat cardiovascular diseases (L. L. Wang et al., 2017). CZTL was a mixed preparation, composed of *Whitmania pigra Whitman* (shuizhi), *Ligusticum chuanxiong Hort.* (chuanxiong), *Salvia miltiorrhiza bunge* (danshen)*, and Astragalus membranaceus* (huangqi). Serval studies reported that CZTL improved neurological function and reduced inflammatory reaction in patients with acute cerebral infarction while reducing the incidence of adverse reactions (Jingwen, 2020; Wang et al., 2021; Min et al., 2022). However, whether the clinical efficacy of CZTL was connected to its influence on microcirculation remained unclear. The present study showed that administration of CZTL preserved microcirculation in AMD rats induced by EHI and ice water. Our findings were consistent with these clinical observations and provided the mechanistic explanation for it.

The microvascular network of patients with ischemic stroke was poorly perfused even if large-vessel occlusion was diminished (Pedersen et al., 2012). It would result in adverse clinical outcomes, thus methods for dynamic *in vivo* monitoring of blood flow were critically required for ischemic stroke patients. Laser speckle contrast imaging was a simple, fast and low-cost imaging method for real-time visualization of blood flow (Senarathna et al., 2013). In the past decades, LSCI was readily accessible for many applications ranging from basic physiology to clinical settings. LSCI was applied to image joints perfusion, burn scar perfusion, skin microvasculature, choroidal circulation, cerebral blood flow, liver and intestine perfusion (Heeman et al., 2019). In this study, the perfusion in the ears, feet and tails of rats were *in vivo* monitored using the LSCI technique, and the degree of microcirculatory damage in rats was assessed. Results showed that the regional blood flow in the ears, feet and tails of AMD rats were decreased compared with healthy rats. Furthermore, CZTL reversed the poor perfusion of ears, feet and tails in AMD rats.

Endothelial cells were identified as a key cell type in the microcirculation (Jackson, 2022). When a vascular lesion occurred, endothelial cells responded immediately and expressed tissue factors, which activated coagulation and led to the formation of thrombin (Verhamme and Hoylaerts, 2006). It was proved that most endothelial functions exhibited the pro-coagulant and anti-fibrinolytic properties during sepsis (Ait-Oufella et al., 2010). It was clear from our results that AMD rats developed exacerbation of coagulation function, which was somewhat ameliorated by ASA and CZTL treatment.

The increased low shear viscosity of rats blood indicated the enhanced red blood cell aggregation (Baskurt and Meiselman, 2003), which resulted in decreased endothelial nitric oxide synthase expression and further impairing tissue perfusion (Baskurt et al., 2004). The abnormally elevated plasma viscosity suggested increased plasma concentrations of high-molecular-weight proteins (mostly fibrinogen), great flow resistance and a decrease of blood flows (Lee et al., 2008). These decreases in regional blood flows contributed to an ischemic/hypoxic environment, leading to endothelial cells damage in micro-vessels (Bai et al., 2010). Abnormal blood rheology existed in subjects with microcirculatory dysfunction and might contribute to the pathophysiology of disease (Lipowsky, 2005). In this study, results of hemorheology showed that ASA and CZTL treatment partially rescued this phenotype, with a reversal of increased blood viscosity in AMD rats. In general, CZTL had significant efficacy in AMD rats including increased local blood flow, reduced blood viscosity and recovered coagulation function.

In addition to coagulation function and hemorheology, microcirculatory function was also demonstrated by the regulation of vascular tone, the capacity to synthesize hemostatic factors and endothelial intercellular permeability. Nitric oxide produced by NOS was a key factor in vasodilation (Hong and Kim, 2017); endothelial intercellular permeability was regulated by adherens junctions (likely VE-cadherin) (Bazzoni and Dejana, 2004); vWF, a hemostatic protein, played a crucial role in regulating angiogenesis and vascular repair (Ishihara et al., 2019). Our results showed that CZTL administration had beneficial effects on microcirculatory function, including promoted vasodilation, enhanced the integrity of the endothelium and increased angiogenesis factor.

In the efficacy evaluation experiment, the low-dose, medium-dose and high-dose groups of CZTL administration were set up. The low dose is calculated according to clinical dose of CZTL and the conversion formula of body surface area, and the medium and high doses are 2 times and 4 times of the low dose respectively. From all indicators, we observed that CZTL had the best effect at medium dose, which may be because the bioavailability of CZTL had reached saturation at medium dose. It will be interesting to explore the pharmacokinetics, *in vivo* clearance and bioavailability of CZTL, which is also the main content of our next work. On the other hand, Ku Yaping et al. found that 3-days pretreatment CZTL inhibited neuroinflammation and oxidative stress in mice with ischemia-reperfusion (Yaping et al., 2021); another study found that pretreatment CZTL for 5 days improved the inflammatory reaction and inhibited apoptosis (Haiyan et al., 2021). Considering the different sensitivity of rats and mice to drugs and the difference of animal models, we chose to pretreat rats with CZTL for 7 days to observe the efficacy of CZTL. Whether prolonging the time of preventive administration or administration after modeling will affect the efficacy of CZTL is a subject worthy of further exploration.

To better understand the metabolic response to CZTL intervention, we performed untargeted metabolomics analysis based on UPLC-Q/TOF MS in rats serum samples. The first finding was that the metabolic status was markedly altered in AMD rats. And then, the present study highlighted a potential that CZTL had a unique role in mediating metabolism. Furthermore, we found that the contents of 55 differentially regulated metabolites were altered in AMD rats and restored by CZTL treatment. Furthermore, KEGG pathway analysis revealed sphingolipid metabolism, arachidonic acid metabolism, glycerophospholipid metabolism, pyrimidine metabolism, linoleic acid metabolism, porphyrin and chlorophyll metabolism, arginine and proline metabolism were significantly influenced by CZTL intervention.

In the sphingolipid metabolism pathway, sphingosine-1-phosphate (S1P) was generated intracellularly from the phosphorylation of sphingosine by sphingosine kinases (SphK) (Ogretmen, 2018). S1P and sphinganine 1-phosphate both were degraded to ethanolamine phosphate via sphinganine-1-phosphate aldolase (SGPL1) (Karuppuchamy et al., 2020). Our results showed that CZTL significantly increased the levels of sphingosine 1-phosphate (*p* = 0.001, FC = 1.94) and sphinganine 1-phosphate (*p* < 0.001, FC = 2.31) in AMD rats. These data predicted that CZTL would increase the level of S1P through repression of SGPL1 rather than activation of SphK. S1P was a key signaling mediator involved in a range of cellular processes, including embryonic development, angiogenesis, immune response and inflammation (Tsai and Han, 2016; Cartier and Hla, 2019). The ligation of S1P to S1P receptor-1 on endothelial cells elicited the enhancement of barrier function by stimulation of Rho- and Rac-dependent assembly of adherens junctions (Shepherd et al., 2017; Abdel Rahman et al., 2021). S1P not only induced the translocation of VE-cadherin to endothelial cell junctions, but also activated N-cadherin to strengthen intercellular interactions (Adamson et al., 2010). Accounting for the increase of VE-cadherin and decreases of IL-6 in CZTL administration rats, a potential mechanism underlying CZTL on microcirculation emerged from coordinated augmentation of junctional integrity and anti-inflammatory responses through sphingolipid metabolism pathway. This underlying mechanism was supported by previous studies: the study by Chen *et al.* indicated that the anticoagulant activity of *Whitmania pigra Whitman* was impacted by the modulation of sphingolipid metabolism (Chen et al., 2021); *Astragalus* polysaccharide was main active ingredient of *Astragalus membranaceus*, and it significantly reversed the disorder of sphingolipid metabolism (Y. Zhang et al., 2021).

**FIGURE 7:**
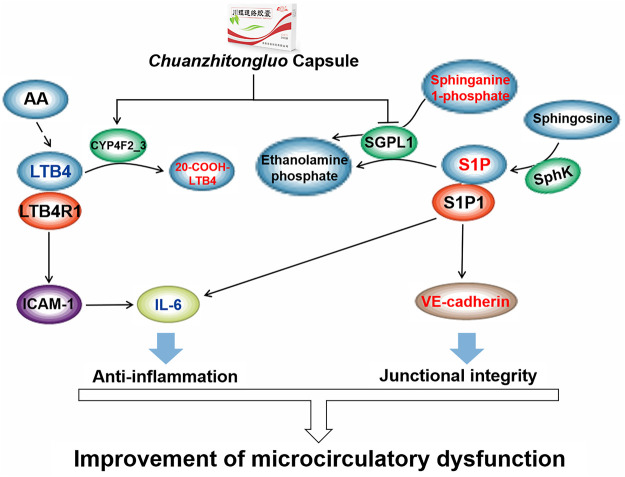
The underlying mechanism of *Chuanzhitongluo* capsule ameliorating microcirculatory dysfunction. AA, arachidonic acid; LTB4, leukotriene B4; LTB4R1, leukotriene B4 receptor type 1; ICAM-1, intercellular cell adhesion molecule-1; IL-6, interleukin-6; SGPL1, sphinganine-1-phosphate aldolase; S1P, sphingosine-1-phosphate; SphK, sphingosine by sphingosine kinases; S1P1, sphingosine-1-phosphate receptor type 1.

20-Carboxy-leukotriene B4 was an omega-oxidized metabolite of leukotriene B4 (LTB4), formed by leukotriene-B4 20-monooxygenase (CYP4F2_3) in the arachidonic acid metabolism pathway. Compared with AMD rats, LTB4 was decreased (*p* = 0.001, FC = 0.61) and 20-carboxyleukotriene B4 was elevated (*p* = 0.047, FC = 1.16) in CZTL medium-dose administration rats. As a result, we would like to propose that CZTL altered the activity of CYP4F2_3 to control the LTB4 content. LTB4 was termed as a potent chemoattractant for leukocyte (Rios-Santos et al., 2003), and it promoted innate immune responses through stimulating the production of other inflammatory mediators (Di Gennaro and Haeggström, 2014). A study in mice showed that LTB4/LTB4 receptor type 1 signaling contributed to LPS-induced hepatic microcirculatory dysfunction by activating inflammatory response (Ito et al., 2008). We observed the increase of proinflammatory factor IL-6 in AMD rats, and it was restored to the normal level with CZTL administration. As reported the literatures, each of medicinal material in CZTL has the function of regulating arachidonic acid metabolism. The *Whitmania pigra Whitman* attenuated blood hyper-viscosity *via* the metabolic reprogramming involved in arachidonic acid metabolism (X. Wang et al., 2019). The bioactive components of *Ligusticum chuanxiong Hort* reduced the release of arachidonic acid and increases of arachidonoyl phosphatidylcholine levels through inhibition of cytosolic phospholipase A (2) (J. Yang et al., 2012). Several studies demonstrated that *Salvia miltiorrhiza bunge* extracts improved arachidonic acid metabolism to exert its efficacy (Park et al., 2008; Xiang et al., 2019). The effect of *Astragalus* polysaccharide on arachidonic acid metabolism was proved in renal fibrosis rats (Ren et al., 2020). Based on our work and literatures, we presented that CZTL might alter the arachidonic acid metabolism and control the LTB4 content to inhibit the excessive inflammatory response and improve the microcirculation function (Figure 7). However, the interaction between CZTL and CYP4F2 or SGPL1 needs further experiments to verify. Whether S1P inhibitor or CYP4F2 agonist can block the efficacy of CZTL is an important issue, which is also a subject we are exploring.

Glycerophospholipid metabolism pathway controlled lyso-phosphatidylcholine (LPC) formation and metabolism. LPC was reported to influence the chemotaxis of microvascular endothelial cell and to induce superoxide overload *via* regulation of extracellular signal-regulated kinase 1/2 and nitric oxide synthase (Murugesan et al., 2003; Choi et al., 2010). Previous studies suggested that disturbed glycerophospholipid metabolism could be regulated by *Salvia miltiorrhiza bunge* and *Astragalus membranaceus* (Liu et al., 2021; M. Y. Zhang et al., 2019), and these studies provided evidences for our results. Pyrimidine metabolism pathway was enriched from 5-methylcytidine,which was a marker of DNA methylation levels, and reprogramming of DNA methylation of p66(Shc) gene rescued microvascular dysfunction (Chouliaras et al., 2012; Streese et al., 2020). Protoporphyrin IX was the main hub of the porphyrin and chlorophyll metabolism pathway (Ajioka et al., 2006). Plenty of reports indicated that zinc-/tin-/cobalt-protoporphyrin IX inhibited heme oxygenase to regulate microvascular function (Ishikawa et al., 2005; Van Landeghem et al., 2009; Cao et al., 2012). However, only few animal experiments showed that *Salvia miltiorrhiza bunge* and *Astragalus membranaceus* regulated pyrimidine metabolism in mice (Xiang et al., 2019; Liu et al., 2021). Our study found that CZTL containing *Whitmania pigra Whitman*, *Ligusticum chuanxiong Hort.*, *Salvia miltiorrhiza bunge* and *Astragalus membranaceus* had an influence on porphyrin and chlorophyll metabolism pathway. In the linoleic acid metabolism pathway, 12,13-epoxy-11-oxo-9-octadecenoic acid (12,13-ep-9-KODE) was one metabolite of 9-KODE synthesized from linoleic acid (Moran et al., 2000). *In vitro* experiment displayed cis-12,13-epoxy-9-octadecenoic acid pretreatment prevented mitochondrial dysfunction in renal proximal tubular cells (Nowak et al., 2004). A recent study noted that *Astragalus membranaceus* reduced the content of linoleic acid and impacted the linoleic acid metabolism in preweaning dairy calves (Ma et al., 2022). Therefore, our prediction that CZTL regulated linoleic acid metabolism to ameliorate microcirculatory dysfunction were credible. The specific mechanism of arginine and proline metabolism effect on microcirculation were unclear, but previous studies suggested the effects of *Salvia miltiorrhiza bunge* and *Astragalus membranaceus* were associated with regulation of arginine and proline metabolism (Ma et al., 2022; M. Y. Zhang et al., 2019). In this study, the proline level of AMD rats was down-regulated and that of CZTL medium-dose administration rats was up-regulated. Serval *in vitro* studies showed that proline induced apoptotic response and mitigated oxidative stress through proline dehydrogenase (Hu et al., 2007; Natarajan et al., 2012). When the microcirculatory dysfunction emerged, endothelial cells displayed increased oxidative stress and advanced apoptosis. It would be interesting to verify if CZTL had an interaction with proline dehydrogenase to dissipate oxidative stress and inhibit apoptosis.

## 5 Conclusion

The current study revealed that microcirculatory dysfunction and metabolic disorder appeared in AMD rats. CZTL significantly improved the function of microcirculation in AMD rats including increased microcirculatory blood flow, rescued the excessive coagulation, reduced blood viscosity and up-regulated NOS, vWF and VE-cadherin expression. The UPLC-Q/TOF MS based metabolomics analysis illuminated the potential mechanism of CZTL influence on microcirculation, involving modulation of sphingolipid and arachidonic acid metabolic pathways to promoted anti-inflammatory programs and junctional integrity. Our findings suggested that CZTL might serve as a potential therapeutic options for the treatment of microvascular disease, and it provided novel insights into the clinical efficacy of CZTL in patients with ischemic stroke.

## Data Availability

The original contributions presented in the study are included in the article/Supplementary Material, further inquiries can be directed to the corresponding authors.

## Abbreviations

LSCI, laser speckle contrast imaging; CZTL, Chuanzhitongluo capsule; AMD, acute microcirculatory dysfunction; ASA, acetylsalicylic acid; EHI, epinephrine hydrochloride injection; APTT, activated partial thromboplastin time; PT, prothrombin time; TT, thrombin time; FIB, fibrinogen; ROI, regions of interest; NOS, nitric oxide synthase; vWF, von Willebrand factor; VE-cadherin, vascular endothelial-cadherin; IL-6, interleukin-6; QC, quality control; UPLC, ultra-high performance liquid chromatography; PCA, principal component analysis; OPLS-DA, orthogonal partial least-squares discriminant analysis; VIP, variable important in projection; SD, standard deviation; FC, fold change; WBV, whole blood viscosity; PV, plasma viscosity; LTB4, leukotriene B4; LPC, lyso-phosphatidylcholine.
